# Wedged Fiber Optic Surface Plasmon Resonance Sensor for High-Sensitivity Refractive Index and Temperature Measurements

**DOI:** 10.3390/s22239099

**Published:** 2022-11-23

**Authors:** Lixia Li, Yuli Li, Xueyang Zong, Linlin Zhao, Penglei Li, Kun Yu, Yufang Liu

**Affiliations:** Henan Key Laboratory of Infrared Materials & Spectrum Measures and Applications, School of Physics, Henan Normal University, Xinxiang 453007, China

**Keywords:** fiber optic sensors, surface plasmon resonance, temperature sensor, micro-optical devices

## Abstract

Here, we experimentally demonstrate a wedged fiber optic surface plasmon resonance (SPR) sensor enabling high-sensitivity temperature detection. The sensing probe has a geometry with two asymmetrical bevels, with one inclined surface coated with an optically thin film supporting propagating plasmons and the other coated with a reflecting metal film. The angle of incident light can be readily tuned through modifying the beveled angles of the fiber tip, which has a remarkable impact on the refractive index sensitivity of SPR sensors. As a result, we measure a high refractive index sensitivity as large as 8161 nm/RIU in a wide refractive index range of 1.333–1.404 for the optimized sensor. Furthermore, we carry out a temperature-sensitivity measurement by packaging the SPR probe into a capillary filled with n-butanol. This showed a temperature sensitivity reaching up to −3.35 nm/°C in a wide temperature range of 20 °C–100 °C. These experimental results are well in agreement with those obtained from simulations, thus suggesting that our work may be of significance in designing reflective fiber optic SPR sensing probes with modified geometries.

## 1. Introduction

Surface plasmon resonance (SPR) is a physical phenomenon that occurs at the metal–dielectric interface when the momentum-matching condition is satisfied for the light wave and the surface plasmon wave. SPR has been widely applied in many fields such as biochemical analysis, food inspection, environmental monitoring, etc. [1,2,3,4,5,6]. Conventional SPR sensing platforms are based on the Kretschmann configuration, where propagating plasmons are excited by compensating the momentum of incident light with a high-refractive-index (RI) prism. Such plasmons have a spatial extension comparable to the light wavelength, leading to very high RI sensitivities for conventional prism-based SPR sensors [7,8]. Nowadays, SPR sensors using the Kretschmann geometry have become a mature technology and are commercially available, whereas their bulky constructions limit in situ monitoring. In the past decade, fiber optic SPR sensors have been demonstrated to be a promising alternative to prism-based sensors due to their many advantages, such as low cost, lightweight, inertness to external electromagnetic interference, and in situ monitoring [9,10,11,12], which provides a new opportunity to miniaturize propagating SPR sensing devices.

One of the most common applications of the fiber optic SPR sensing system is temperature monitoring, which has been reported extensively in recent years [13,14]. For instance, SPR temperature sensors have been reported based on various fiber structures, including U-shaped fiber [15,16], D-shaped fiber [17,18], internally filled hollow fiber [14,19], spliced fiber [20,21], etc. The above sensors have been shown to have good sensing performance, but their detection method is transmission type, which limits the application of the sensing system. Moreover, the sensing sensitivity can be further improved. Reflective fiber optic SPR probes with modified geometry at the end of the fiber have been developed [22,23,24,25], which are in line with the modality of prism-based SPR sensors. In this kind of sensor, the SPR coupling wavelength can be readily controlled by modifying the fiber tip geometry in order to achieve the highest performance within a limited wavelength range. More importantly, the sensing probe allows the sensor to be easily immersed into compact spaces and provides more degrees of freedom to engineer versatile sensing devices.

Here, using common multimode fibers (MMF), we experimentally devise a strikingly simple sensing platform—wedged fiber optic SPR sensing probe. Through optimizing the beveled angle of the fiber tip and encapsulating it with n-butanol, we assess the temperature-sensing performance of the sensor and verify that the device working at the visible and short-wavelength near-infrared regions (400 nm < *λ* < 1000 nm) can detect a wide temperature range with high sensitivities. These experimental results are well verified by simulations.

## 2. Methods

**Figure 1:**
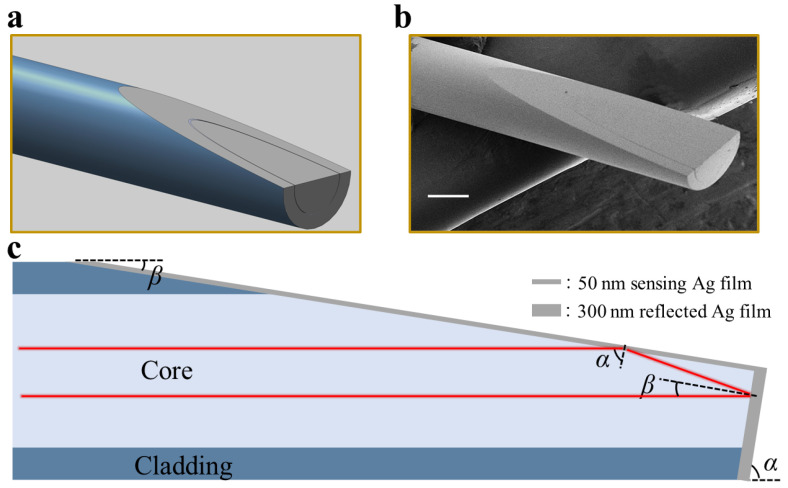
(**a**) Schematic view of the wedged fiber optic SPR probe. (**b**) Scanning electron microscope image of the fabricated probe. Scalebar: 200 μm. (**c**) Definition of involved parameters of the sensing probe.

Figure 1a shows the schematic diagram of the designed fiber optic SPR probe, and Figure 1b shows the scanning electron microscope image of the prepared probe that was experimentally tested. Here, the step-index MMF with 400 μm core diameter and 0.22 numerical aperture was used, where the RI of the fiber core was 1.457. The fiber tip was polished into a wedged geometry with two asymmetrical bevels, and the intersection of the two bevels was at the center axis of the fiber optic, shown in Figure 1c, with the angle between the two bevels kept at 90°. The bevel with the angle of *β* = 9° was coated with a 50-nm-thick silver film, which acts as the sensing region, and the other bevel was coated with a 300-nm-thick silver film as a reflected mirror. The white light source was coupled to the sensing region with the angle of *α* and was reflected from the mirror with the angle of *β*, where *α* and *β* are the two polishing angles and satisfy *α* + *β* = 90°.

When the light incident on the interface is between the fiber core and silver film, the evanescent wave is generated and propagated into ambient medium. The component of the wave vector is:

(1) $$K_{z}=\frac{2\pi }{\lambda }\sqrt{\varepsilon_{0}}sin\alpha$$

where λ is the wavelength of the incident light, $\varepsilon_{0}\;$ is the dielectric constant of the fiber core, and *α* is the incident angle.

At the interface between the medium and silver film, the plasma oscillation confined to the metal surface produces an electromagnetic wave propagating in the *Z*-direction, and its amplitude is attenuated exponentially in the *Z*-direction, which is called the surface plasmon polariton (SPP). The propagation constant of SPP is:

(2) $$K_{spp}=\frac{2\pi }{\lambda }\sqrt{\frac{\varepsilon_{m}\varepsilon_{d}}{\varepsilon_{m}+\varepsilon_{d}}}$$

where $\;\varepsilon_{m}$ is the dielectric constant of the metal film and $\varepsilon_{d}\;$ is the dielectric constant of the medium. When $\;K_{spp}=K_{z},\;$ the incident light energy is transferred to the surface plasma wave so that the surface plasma wave can be excited.

**Figure 2:**
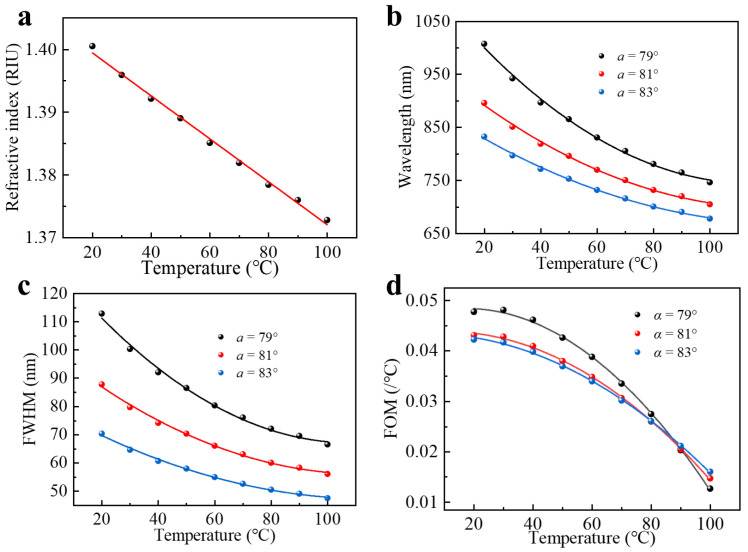
(**a**) Variation of RI of n-butanol with temperature. (**b**) Simulated SPR wavelengths. (**c**) Spectral FWHM and (**d**) spectral FOM as a function of temperature under different fiber polishing angles. The nonlinear fitting curves are to guide the eyes.

Experimentally, we use n-butanol (content ≥ 99.5%) as the thermally responsive material to carry out the ambient temperature measurement. In order to obtain the thermal optical coefficient (TOC) of n-butanol that characterizes the change in the RI of optical materials with the ambient temperature, we employed a traditional fiber optic SPR sensor with a well-defined robust sensitivity model to calibrate the RIs when the ambient temperature changed from 20 °C to 100 °C. As shown in Figure 2a, there is a significant negative linear correlation between the RI and temperature. Using linear fit, the RI of n-butanol as a function of temperature can be expressed as:

(3) $$n\left(T\right)=-3.31005\times 10^{-4}T+1.40543$$

with *T* being the ambient temperature and the TOC = −3.31005 × 10^−4^ RIU/°C. The TOC is a factor that has an active impact on temperature sensitivity. As a result, the quest for optical materials with high TOC is important for enhancing the performance of SPR temperature sensors. Additionally, the fact that SPR sensors themselves possess high RI sensitivities can improve the response of devices to temperature. Typically, as the SPR coupling wavelength increases, the RI sensitivity of SPR sensors based on wavelength modulation can increase rapidly. Therefore, to obtain a high-temperature sensitivities of the wedged fiber optic sensor device, we can increase its resonance wavelength as far as possible in a limited spectral region. The resonance wavelength can be tuned by controlling the incident angle *α*. Here, we calculated the reflection spectra of the fiber optic SPR sensor using the finite element method. We choose the sensing silver layer of 50 nm for our simulation, the RI of fiber core was set to 1.457, the dielectric constant of the silver film was determined by the Drude–Lorentz model [26], and only the situation in which the light from the meridian surface excites the SPR was considered in the analysis of the sensing model. The position of peaks as a function of temperature for three incident angles is shown in Figure 2b. One can clearly see that as the incident angle decreases, the resonance wavelength redshifts, and the temperature sensitivity has a significant increase. However, for the angle of *α* = 79°, the SPR wavelength exceeds the detection range (326–1035 nm) of our spectrometer, and, on the other hand, the corresponding spectral full width at half maximum (FWHM) broadens obviously (shown in Figure 2c), which affects the final accuracy of the peak tracking. Moreover, the sensitivity and figure of merit (FOM) is a necessary parameter to evaluate the SPR sensor performance, which can be expressed as:

(4) $$FOM=\frac{\left|S\right|}{FWHM}\;$$

where the *S* is defined as the sensitivity of the SPR sensor. As shown in Figure 2d, in most temperature ranges, the FOM of the SPR spectrum at 81° was higher than at 83°. To achieve the highest possible FOM in the detection range, we finally chose the incident angle of *α* = 81° for our experiments.

## 3. Experiments and Discussion

**Figure 3:**
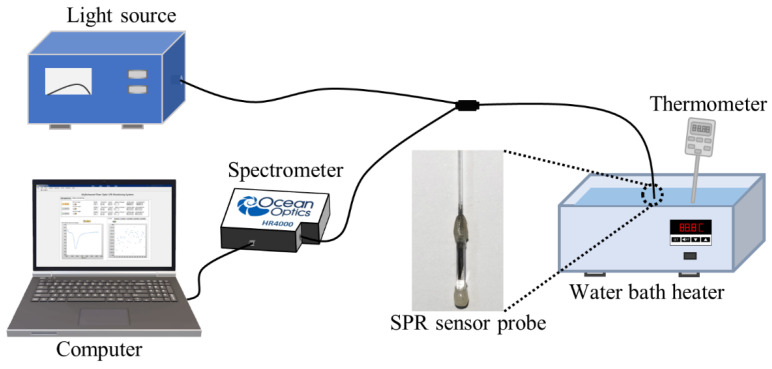
The sketch diagram of the sensing system. Inset: The optical fiber SPR temperature-sensing probe.

After obtaining the appropriate polishing angles, we fabricated the sensing probe with the following steps. First, the fiber tip was polished to a bevel with angle of *α* = 81° by a fiber optic polishing machine (Ultrapol5, Ultrapol, Santa Ana, CA, USA), and the other bevel was produced with an angle of *β* = 9°. Second, the magnetron sputter coater (Q300TD plus, Quorum, Laughton, United Kingdom) was employed to coat the metal film onto the fiber tip. The bevel with angle of *α* = 81° was coated with 5 nm-thick chromium and 300 nm-thick silver film, and the other bevel was then coated with 5 nm-thick chromium and 50 nm-thick silver. The chromium film was mainly to increase the adhesion between the silver film and the fiber optic. The film thickness was monitored by a crystal oscillator. Lastly, we used a 1 cm-long glass capillary to package the fiber tip. One end of the glass capillary was sealed with epoxy AB glue. After the glue was cured and positioned, n-butanol was filled into the glass capillary with a syringe. The sensor probe was fully encapsulated and placed for 24 h to allow the glue to reach its highest strength. The structure of the package could effectively protect the metal layer from oxidation and damage and increase the reuse rate of the sensor. The prepared fiber optic sensing probe is shown in the inset of Figure 3.

Figure 3 shows the sketch diagram of our temperature experimental system. In this measurement, broadband light from a halogen lamp (HL-2000, Ocean Optics, Dunedin, FL, USA) was launched into the fiber probe by a Y-type fiber splitter, which and excited an SPR effect in the sensing region. The light was then reflected into the fiber splitter by the reflected mirror of the wedged fiber optic probe and received by an optical fiber spectrometer (HR4000, Ocean Optics, Dunedin, FL, USA). The spectrometer was connected to the computer via a USB interface, and the experimental data were collected and processed in real time using a self-made Labview program. The sensing probe was dipped in the water bath heater (BHS-2, Joanlab, Huzhou, Zhejiang, China) during the temperature-up period, and a thermometer was used to monitor temperature.

**Figure 4:**
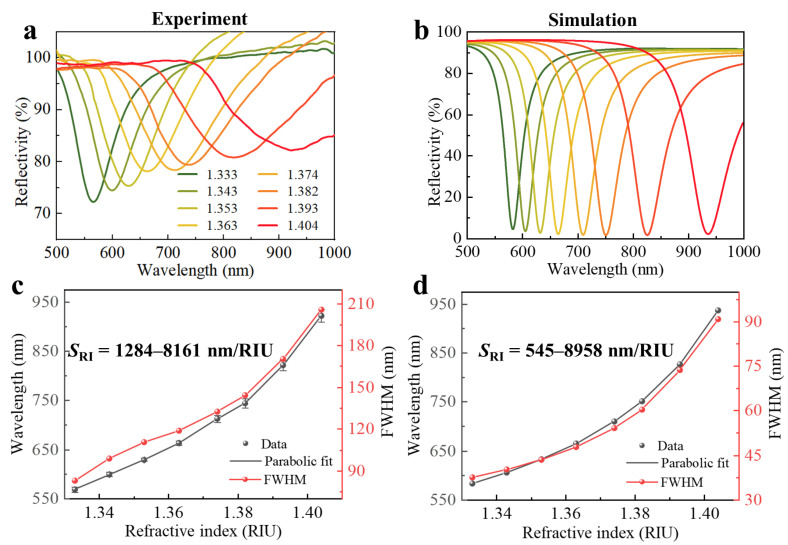
(**a**) Experimental and (**b**) simulated reflection spectra for different RIs. (**c**,**d**) Resonance wavelengths and spectral FWHM as a function of RIs, which correspond with panels (**a**) and (**b**), respectively.

The RI response of the fiber optic SPR sensor was examined first. The fiber probe was immersed into a series of refractive index liquids with the range of 1.333 to 1.404. As shown in Figure 4a, as the RI of the solution increased from 1.333 to 1.404, the resonance wavelength was red-shifted from 568.852 nm to 922.387 nm. Here, we used the bulk RI sensitivity, *S_RI_* = Δ*λ*/Δ*n*, to quantify the sensing performance of the sensor, where Δ*λ* is the shift in the resonance wavelength and Δ*n* is the change in the environment refractive index. The resonance wavelength with respect to RI and experimental spectral FWHM are displayed in Figure 4c. The error bars were determined as the maximum measured standard deviation of three RI measurements and indicate that the repeatability of the sensor was within an acceptable range. We fit the experimental data to a polynomial formula (a solid line) and obtained the following equation:

(5) $$\lambda =48426.3n^{2}-127820.4n+84913.25$$

From the equation, the fiber optic SPR sensor was found to have the RI sensitivity S_RI_ = 1284–8161 nm/RIU for n = 1.333–1.404. Our experimental resonance wavelength results are strongly in line with those of simulations, as shown in Figure 4b,d, and the change trend of spectral FWHM is the same as that of simulations.

**Figure 5:**
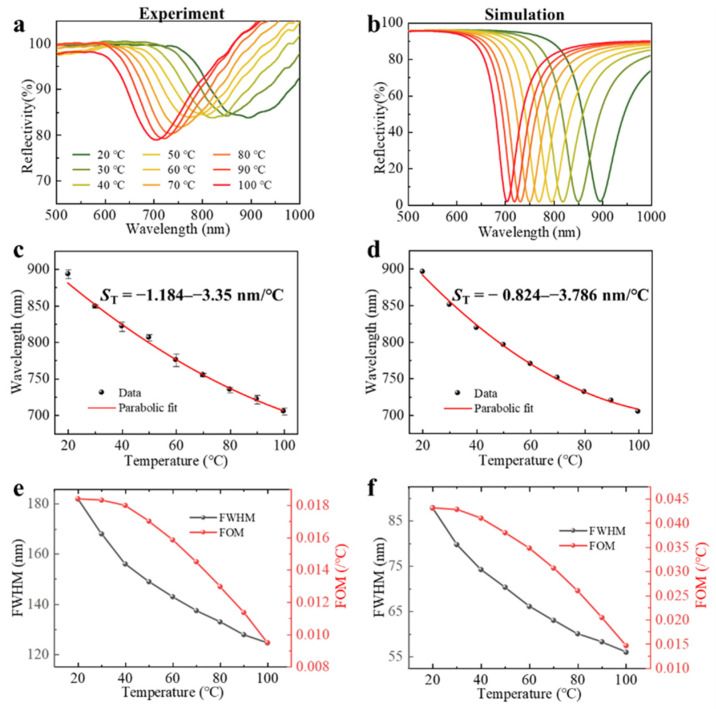
(**a**) Experimental and (**b**) simulated reflection spectra for different temperatures. (**c**,**d**) Resonance wavelengths as a function of temperatures, which correspond with panels (**a**) and (**b**), respectively. (**e**) Experimental and (**f**) simulated spectral FWHM and FOM change with temperature.

Next, the sensing probe packaged with n-butanol was immersed in the water bath heater, and the change in resonance spectrum when the temperature rises from 20 °C to 100 °C with the increment of 10 °C was observed. Considering the accuracy of the measurement, each temperature point needed to be preheated for one minute and kept for three minutes before the next measurement. Figure 5a shows the SPR spectra under different temperatures. We observed that as the temperature rose from 20 °C to 100 °C, the resonance wavelength showed a clear blue shift from 893.641 nm to 705.453 nm, which was the result of the decrease in the refractive index of n-butanol as the temperature increases. As illustrated in Figure 5c, the maximum measured standard deviation of the three temperature measurement values did not exceed ±6.353 nm. The experimental data were fitted to a polynomial formula (a solid line) that can be expressed as:

(6) $$\lambda =0.01354T^{2}-3.8921T+960.752$$

which demonstrated that the fiber optic SPR sensor had the temperature sensitivity S_T_ = −1.184–−3.35 nm/°C for *T* = 20 °C–100 °C. The temperature simulation results are shown in Figure 5b,d, where it can be seen that the sensor has good temperature-sensing performance in both experiments and simulations.

Moreover, as shown in Figure 5e, in the temperature range of 20 °C to 100 °C, the experimental spectral FWHM was variable in the range of 125 nm to 182 nm, and the FOM was up to 0.018/°C. The FWHM and FOM of the simulated temperature spectra are also depicted in Figure 5f. There was a close agreement between the experimental and simulated results.

As shown in Table 1, compared with other recently reported fiber optic sensors, the wedged fiber optic SPR sensor proposed in this paper had a high RI and temperature sensitivity [10,11,12,14,17,20,21,22,23]. Moreover, the structure of the sensor is compact, which is more conducive to the practical integration of sensors.

## 4. Conclusions

In summary, a compact wedged fiber optic SPR sensor probe with adjustable incident light angle was experimentally verified by polishing an MMF into a wedged structure with two asymmetric bevels at the tip and coating it with Ag film. After being optimized for the beveled angles, the sensor probe exhibited a high RI sensitivity as large as 8161 nm/RIU. The temperature sensitivity could reach up to −3.35 nm/°C after being packaged into the n-butanol solution with high thermal optical coefficient. Moreover, the experimental results of the sensor are consistent with the simulation results. The proposal of the fiber optic SPR sensor may provide more possibilities for designing reflective fiber optic SPR sensors with modified geometries.

## Figures and Tables

**Table 1:** Comparison of the proposed sensor with other sensors’ performance.

Sensor Type	S_RI_ (nm/RIU)	RI Range (RIU)	S_T_ (nm/°C)	T Range (°C)	FWHM (nm)	FOM (/°C)	Sensitization Method
Unclad MMF [10]	4358.4	1.36–1.41					Cu/ITO
Tapered fiber SMF [11]	4166.7	1.358–1.410	—	—	—	—	Al/TiO_2_
D-shaped SMF [12]	2765	1.410					Au
U-shaped MMF [14]	—	—	−0.978	25–100	79–83	0.012	Au/UV-curable
Internally filled hollow fiber [17]	—	—	−1.16	35.5–70.1	75–123	0.009–0.033	Ag/alcohol
Spliced MMF-PCF-MMF [20]	—	—	−1.551	35–100	—	—	Au/PDMS
Circular truncated cone-shaped twin-core fiber [22]	—	—	−2.07–4.13	20–70	70–121	0.029–0.035	Au/PDMS
Pencil-shaped MMF [23]	—	—	−1.985	20–70	98–142	0.014–0.02	Ag/PDMS
This Paper	1284–8161	1.333–1.404	−1.184–3.35	20–100	125–182	0.01–0.018	Ag/n-butanol

## Data Availability

Not applicable.
